# An Exploration of Intent to Use Telehealth at Home for Patients with Chronic Diseases

**DOI:** 10.3390/ijerph14121544

**Published:** 2017-12-09

**Authors:** Shu-Lin Uei, Yu-Ming Kuo, Chung-Hung Tsai, Yu-Lun Kuo

**Affiliations:** 1 Department of Telecare, Mennonite Christian Hospital, Hualien City 970, Taiwan; mchwsl@gmail.com; 2 Department of Marketing and Distribution, Tzu Chi University of Science and Technology, Hualien City 970, Taiwan; ss248@ems.tcust.edu.tw; 3 Department of Health Administration, Tzu Chi University of Science and Technology, Hualien City 970, Taiwan; tsairob@tcust.edu.tw; 4 Department of Nursing, Tzu Chi University of Science and Technology, Hualien City 970, Taiwan

**Keywords:** telehealth, chronic disease, self-health management, eHealth

## Abstract

Telecare is defined as care practiced at a distance. It is an effective strategy for improving the self-health care management of home-patients with chronic diseases. The purpose of this study was to explore the intent to use of telehealth patients. The correlation between the self-care behaviors, the intent to use of telehealth, and the effects on physiological indicators of patients with chronic disease at home were studied. A cross-sectional study design employing purposive sampling was selected. The structured questionnaire ‘Telecare Usage Intention Scale and Self-Care Behavior Scale’ were used, ‘HbA1c, glucose levels and monthly blood pressure measurements’ were analyzed in this thirteen month study. The self-care behaviors of the participants were positively correlated with their intent to use telehealth (*p* < 0.01). The results also indicated that HbA1c, glucose levels and frequency BP measurement of the participants improved significantly after using telecare (*p* < 0.005). The results indicated a strong intent to use telehealth and positive perception of telecare services by in-home patients with a chronic disease. Telehealth improves the self-care behavior of in-home chronic disease patients and enhances medical professionals’ ability to deliver quality and effective healthcare.

## 1. Introduction

### 1.1. Background

Non-communicable diseases (NCDs), also known as chronic diseases (CD), such as asthma, chronic obstructive pulmonary disease (COPD), diabetes, heart failure and hypertension, affect millions on a global basis [1]. The number of NCD deaths has increased worldwide, from 38 million in 2012 and is projected to reach 52 million by 2030 [2]. On a global basis, the toll of diabetes alone accounted for 1.5 million deaths. It was the eighth leading cause of death for both genders and the fifth leading cause of death in women in 2012. It is estimated by the WHO, that 422 million adults aged over 18 years were living with diabetes in 2014. Approximately half the diabetes cases in the world can be found in South East Asia [3]. In Taiwan, the 10 leading causes of death in 2015 were NCDs: for which diabetes and hypertension were listed as the fifth and eighth most frequent causes of death [4]. The annual medical expense incurred by patients with NCD in Taiwan is almost NT$70 billion and continues to rise. Between 2004 and 2014, the annual medical expense for treating diabetes patients increased by approximately NT$6.2 billion [4]. Patients with NCD require long-term, continuous care. The existing equipment, treatment procedures, and short-term care approaches employed by the medical system for acute diseases do not meet the requirements of patients with NCD [5]. The existing care approaches for patients with NCD require significant improvements.

In response to the multifaceted care requirements of patients with NCD, the medical personnel and scholars from the U.S. MacColl Institute co-developed a chronic care model. It proposes six mandatory elements for long-term care: (1) the organization of health care; (2) delivery system design; (3) decision support; (4) clinical information systems; (5) self-management support; and (6) resources and policies [6]. Particularly vital for improving the health management and self-care capabilities of patients with NCD, is self-management support. Patients with NCD require long-term and continuous medical intervention and assistance from others. Additionally, due to the changes in traditional Asian familial social structure and the limited number of hospitalization days covered under the National Health Insurance (NHI) system, NCD patients are often discharged early and forced to rely on their communities or home for further health care [7]. Therefore, patients with NCD living at home, in communities, or in institutions must implement appropriate self-care and health management practices to effectively control their diseases and prevent comorbidity [8].

Telecare is defined as care practiced at a distance. It is an effective strategy for improving the self-care capabilities of patients with NCDs. With the advent of information communication technology utilized to provide patients with NCD medical services; technologies such as telephones, mobile phones, emails, instant messages, and online video calls are used for service delivery or communication between medical professionals and NCD patients [9]. Currently six telecare systems exist: (1) home tele-monitoring; (2) clinical decision-making and instant warning response; (3) NCD self-management education; (4) periodic care services; (5) resource links and referrals to other medical teams or institutes; and (6) remote video conferences. Home tele-monitoring, the most prevalently applied telecare form, involves installation of self-monitoring devices in the homes of patients with NCDs. This allows the medical team to monitor the patients’ physiological, psychological, or behavioral indicators and transmit patient data to remote monitoring centers. This data enables health professionals in evaluating patient health statuses and allows for rapid medical response [10]. Thus, NCD patients enable the monitoring of their own physiological indicators, self-manage their health, and acquire appropriate health education.

A literature review revealed that most studies report that telecare had positive effects and almost none reported negative effects on CD management [11]. Telecare led to patients and families taking a more active role in disease self-management. Patients monitored by telecare showed significant improvement in glycemic control in type 2 diabetes when compared with those monitored by routine follow-ups [12]. Telecare profoundly benefits patients with NCDs, by contributing toward their health management, enabling them to preemptively identify and mitigate their diseases, reducing the frequencies of clinical visit, and shortening the time of their clinical visits. As a result of these benefits, Telecare acts to reduce the cost of healthcare [13]. These benefits demonstrate the effectiveness and importance of telecare for patients with NCDs.

Currently, most studies on telecare in Taiwan have focused on the establishment of information platforms and systems or scales measuring the effectiveness of Telecare: few have explored the intent to use telehealth (IUT) from the patients’ perspective. Patients’ requirements for and acceptance of Telecare has been explored in the research of Tai et al. [5] and Lin et al. [14], along with the benefits of these services. It is a strongly recommended care model for patients with NCD in Eastern Taiwan, which is characterized by its rough terrain and remote mountain areas.

### 1.2. Purpose

The purpose of this research was to explore the IUT for in home diabetes and/or hypertension patients. The resulting data was analyzed to determine intent to use of Telehealth and any correlation between the IUT and the self-care behaviors of in-home CD patients. The focus of the purposive sampling within the domain of NCD to satisfy the goals of the research and hospital program effectiveness was diabetes and hypertension as both diabetes and hypertension may be monitored accurately with technology currently available to patients.

## 2. Methods

### 2.1. Study Design

This was a cross-sectional research. Through purposive sampling, patients with diabetes and/or hypertension who had used the health management platform, Personal Health Records (PHRs), from the telecare center of a hospital in Eastern Taiwan were selected. Linking the telecare medical unit (Gateway, Yi Yuan Medical Technology Company, Taipei, Taiwan) to the existing landline telephone or other network allowed for convenient integration of the health care equipment within the patient’s home environment. Telecare as a long distance intervention allows for managing patients PHR, monitoring medical treatment, and health education, while tracking patients’ statuses of their disease self-care management. Autonomy of the participant in controlling their state of health is promoted through telephone communication from a member of the medical staff at the PHR. A structured questionnaire, using purposive sampling was conducted to examine the IUT of the telecare service and correlations between the IUT and self-care behaviors was examined to further improve future development of telecare services. Additionally, specific benefits of the telecare intervention were investigated through analysis of the patients’ physiological and psychological indicators. This study was conducted from 1 April 2014 to 31 March 2015.

### 2.2. Participants

Participants were purposively selected from a telecare center of an Eastern Taiwan hospital. Research subjects were requested to participate in the PHR by the diagnosing doctor, as most of these patients live in remote or extremely rural areas. Selection criteria was as follows: (a) hypertension, diabetes, or both diseases were diagnosed; (b) not younger than 20 years old and (c) communicate in Mandarin Chinese or Taiwanese. The exclusion criteria were as follows: (a) having a psychiatric or other disease that could affect cognitive capabilities, or prevent him or her from answering the questionnaires; (b) refusal to participate in the study. This study received approval from the Institutional Research Board (IRB14-07-012) after adjustments were made to the questionnaire according to their guidelines. Consent was requested and obtained from all participants in written form. Privacy of the participants, and any data collected in this study are treated as strictly confidential and used for academic research purposes only.

### 2.3. Data Collection

The structured questionnaires employed in this study were the telecare usage intention scale (TUS), the self-care behavior scale (SCS), and participant demographic information. Face-to-face interview was the data collection method of preference. In addition, the physiological indicators included glycated hemoglobin (HbA1c), pre-meal glucose levels pre- and monthly blood pressure (BP) checks. Prior to tele-monitoring, the participants’ glucose levels and BP were measured by medical professionals through the rural medical service or outpatient clinic.

#### 2.3.1. Telecare Usage Intention Scale

The TUS was a revision of the translated telecare scale formulated by Tsai and Chuang [15]. This scale was designed to assess the usage intention of telehealth of patients. The modified scale utilized in this study included 15 items, which covered three dimensions: (1) personal benefits gained from using telecare; (2) the activeness of an individual in using telecare; and (3) the individual perception of telecare. Each dimension comprised five items, which contained three answers, disagree (=1), neutral (=2), and agree (=3). A higher score indicated that the IUT of a participant was greater. The Cronbach’s α value was 0.795.

#### 2.3.2. Self-Care Behavior Scale

The self-care behavior scale was a revision of the self-care behavior scale created by Tseng [16] for elderly people with CDs It was designed to evaluate self-care behaviors of participants at home. This modified scale comprised 13 items, covering three dimensions: (1) medication compliance behaviors (four items); (2) self-care behaviors (five items); and (3) health promotion behaviors (four items). Each item contained five answers: never (=1), rarely (=2), occasionally (=3), frequently (=4), and always (=5). The maximal and minimal scores of the scale were 65 and 13 points respectively; the higher the score the more satisfactory the self-care behavior of the participant. The Cronbach’s α value was 0.873.

#### 2.3.3. Demographic Information

The demographic information investigated in this study included the patients’ sex, age, marital status, education level, occupation, area of residence, CD history, and medication status.

#### 2.3.4. Physiological Indicators

The physiological indicators examined in this study were the HbA1c and AC glucose levels and the monthly BP check. Physiological data was collected pre and post one month of the study’s completion to allow for analysis of change in physiological parameters.

### 2.4. Data Analysis

The Statistical Package for the Social Sciences Version 21.0 for Windows (IBM, Armonk, NY, USA) was employed for data archiving, descriptive and inferential statistical analyses. The data was converted into percentages to clarify the structural distribution of the participants’ demographics. Descriptive statistics were applied to describe the IUT by the participants. Pearson correlation analysis was conducted to explore the correlation between the self-care behaviors of the participants and their IUT. A general linear model was used to examine the factors that affected the IUT of the participants. A paired *t* test was used to explore the changes in the participants’ physiological indicators before and after they used the telecare services. The Cronbach’s α value was 0.05; *p* < 0.05 indicated a statistically significant difference.

## 3. Results

A total of 100 patients with CD who had used PHR from a hospital in Eastern Taiwan were surveyed. There were three patients who were unable to answer the questionnaires, two patients that died, three patients who moved to other areas, and 10 who refused to participate in this study were excluded. As such, a total of 82 patients were recruited. Their data analysis is described as follows.

### 3.1. Demographics

The demographics of the patients’ included: sex, age, marital status, education level, occupation, area of residence, CD history, and medication status (see Table 1).

### 3.2. Descriptive Data

#### 3.2.1. Analysis of the IUT of the Participants

More than half of the participants stated that the telecare services were beneficial and accepted the use of the services. However, the participants were less inclined to upload or employ the platform information; more than half of the participants did not proactively upload their dietary (73.2%) and exercise records (75.6%). Additionally, most of the participants (52.4%) felt that telecare services should be paid for by the National Health Insurance (NHI) (Table 2).

#### 3.2.2. Analysis of the Correlation between the Self-Care Behaviors of the Participants and their IUT

The correlations among the self-care behaviors of the participants and their IUT are listed in Table 3. The self-care behaviors of the participants were positively correlated with both their IUT (*p* < 0.01) and the three dimensions of IUT. Those dimensions defined as: (1) the personal benefits gained from using the telecare services (*p* < 0.05); (2) cooperation in uploading or using the platform information (*p* < 0.05); and (3) the participants’ perception of the Telecare services (*p* < 0.05). Consequently, a higher score for self-care behaviors indicated that the IUT of a participant was greater. Additionally, the health promotion behaviors of the participants were positively correlated with the personal benefits gained from using the *t*elecare services (*p* < 0.01) and the participants’ teamwork in uploading and employing the platform information (*p* < 0.01). Correspondingly, the higher a participant scored on health promotion behaviors, the more the participant acknowledged the benefits that the *t*elecare services provided. 

### 3.3. Outcome Data

#### 3.3.1. Factors that Affected the IUT of the Participants

A general linear model was incorporated to investigate the factors that affected the IUT of the participants. The results indicated that the self-care behaviors of the patients were significantly correlated with their IUT (*p* = 0.004) but not with their demographic characteristics. Of the factors that affected each dimension of the IUT, the self-care behaviors of the participants significantly influenced their personal benefits gained from using the Telecare services (*p* = 0.028), their cooperation in uploading or employing platform information (*p* = 0.005), and their perception of the telecare services (*p* = 0.017). The participants’ monthly BP checks significantly influenced their perceived personal benefits gained from using the telecare services (*p* = 0.003). The marital status of the participants significantly affected their consistency in uploading and employing platform information (*p* = 0.028). The results indicate it may require a longer time interval to motivate and empower the majority of patients. The mere provision of telecare services to CD patients is insufficient to maximize its’ potential. The mechanism of empowerment remains a psychologically complex problem. Multiple improvements to telecare services are required as part of continued development. If implemented correctly this should have a positive effect on patient IUT. The correlation analyses support this supposition.

#### 3.3.2. Results of the Physiological Indicators

The physiological indicators examined in this study were the participants’ HbA1c and AC glucose levels as well as monthly BP recording. The analysis indicated that the HbA1c and AC glucose levels of the participants substantially improved after their use of the telecare platform (*p* < 0.005), and their frequencies of BP measurement also increased (*p* < 0.001). See Table 4 for further details.

## 4. Discussion

### 4.1. Effect of Demographic Characteristics on the IUT

Of the participants that were surveyed in this study, 40 were men and 42 were female. The age range of telecare users was 40–64 years old (63.4%) and 30% were older than 65 years. This was consistent with the findings by Lin et al. [14] The usage rate for the over 65 year old participants was much lower than that of the 40 to 64 year old group. This may be due to lack of familiarity using advanced technology products. This finding supports the research of [17] which indicated that the age of patients with CD significantly influenced their IUT of telecare. The bulk of telecare users were married (80.5%) which is consistent with the findings of Lu et al. [18] Married patients with CD used telecare more frequently than did unmarried patients with CD as spouses encouraged and supervised each other’s use of Telecare.

Most of the telecare users did not have education greater than high school (85.4%). This was consistent with the findings by Hong and Deci [19,20], in which the proportions of CD users with education levels no higher than high school were 83.3% and 74–87% respectively. This indicates low education levels amongst most telecare users researched to date. This attributes to the negative correlation between the education levels of the participants and the number of CDs diagnosed (*r* = −0.238, *p* < 0.05). Essentially, as education level lowers, the number of diagnosed CDs increases, which indirectly furthers the related IUT. This suggests that health conditions of CD patients are a factor that affects the healthcare service usage of patients. The greater the CDs a patient has, the higher the rate of the patient’s healthcare service usage.

The demographic characteristics of the participants affects observed telecare usage behaviors. This is explained through the use of the health service utilization model devised by Deci [21] According to this model, three factors affect the healthcare service usage behaviors of patients with CD: (1) predisposing characteristics (e.g., age, sex, marital status, education level, occupation, and religious belief); (2) enabling characteristics (e.g., sufficiency and accessibility of medical resources and the travel time for clinical visits); and (3) needs characteristics (e.g., self-perceived health status and disease status). According to Deci [21], although predisposing characteristics do not directly affect the healthcare service usage behaviors of patients with CD, they directly influence the intentions of the patients to use the services. The usage behaviors of patients vary with patient demographic characteristics. Therefore, on the basis of the analysis in this study, Telecare services should be provided to patients with CD based on their IUT.

### 4.2. Effect of Telecare on in-Home CD Patients

Almost 70% of in-home patients with CD, affirmed the beneficial effect of telecare on their individual health management. This was consistent with the findings of Andersen [22], in which nearly 90% of patients with hypertension at home expressed satisfaction with the convenience and benefits of Uei; Tsai et al. [13] indicated that patients diagnosed with metabolic syndromes were extremely satisfied with the decrease in their health expenditures, revealing a considerably favorable perception of Telecare by patients with CD at home.

The IUT of at-home patients with CD was positively correlated with their self-care behaviors as well as blood glucose and hypertension measurement behaviors. This was consistent with the findings of Andersen [22], in which approximately 80% of telecare users affirmed the benefits of telecare: It was concluded that telecare improved their daily living habits, care knowledge, mitigated worry of personal health status, and promoted interaction with medical professionals. In addition, the participants were more cooperative regarding checking their BP toward the conclusion of the IUT study. This is consistent with the findings of Hong et al. [19], in which hypertension patients uploaded their data more frequently after they began using telecare. Additionally, cognitive, attitudinal, and self-care behaviors improved substantially amongst participants. Similarly, participants showed increased awareness and cooperation regarding monitoring their BP.

This study incorporated portable PHRs, assisting patients in monitoring their daily physiological changes and their own health status. The case managers of the patients then further assessed the health status of each patient and promptly provided health education feedback accordingly; thus improving the patients’ intention and sense of responsibility in caring for their own health. Even though medical professionals reduced the frequency of their interactions with patients, they remained successful in promoting the self-care behaviors of the in-home CD patients.

Telecare motivates patients with CD to learn how to manage their diseases, practice self-care behaviors, become responsible for their own health, and thereby successfully perform self-management of their health. The study of Chen et al. [23] addressed three major tasks of CD self-management: (1) medical management (i.e., taking prescribed medication, exercising, and changing diets according to physicians’ instructions); (2) role management (i.e., adjusting daily living lifestyle to maintain the integrity of the patient’s own daily roles); and (3) emotional management (i.e., learning to manage the psychological stress and negative emotions caused by diseases). Telecare empowers in-home CD patients to alter their behavior, as it makes them aware of their health status and enhances their intrinsic motivation to self-care. The physical presence of the medical technology at their fingertips may act as a visual stimuli to remind patients of their responsibility to themselves. This is consistent with the perspective of self-determination theory [24], in which human behaviors are generated from intrinsic motivation, and that the expression of these behaviors is affected by extrinsic factors.

Unfortunately, the participants showed low cooperation in uploading their dietary and exercise records or in using the health education information from the platform. This may be due to the fact that 95% of the participants used cable telephones for the telecare services, which compromised the ability of uploading dietary and exercise data. Likewise, the health education was primarily provided through telephone inquiries by the case managers. The study by Lo et al. [25] indicated that patients with CD are typically concerned about privacy, telecare usage capabilities, and budget deficiencies, which inhibits their use of telecare services. In this study, 52.4% of the patients felt that telecare should be paid for by the NHI, revealing budgetary considerations as a key factor affecting the future prevalence of telecare services. Telecare mitigates the inequality in medical care caused by distance and time constraints. It also improves the timeliness of health self-management, yet comprehensive consideration is required to maximize the benefits of telecare. Factors such as: (1) suitability of users; (2) costs; (3) service convenience; (4) demands on self-care capabilities; (5) learning motivations of users; and (6) user privacy guarantees, must be incorporated to develop effective Telecare systems within varying local conditions. Due to the cost of telecare, a minority of the participants would not use it, as they feel it should be covered under NHI. This does not suggest that they would reject telecare outright, as the “unwillingness to pay” may be outweighed by the need receive timely medical attention.

In accordance with existing laws, the home-telecare model developed in this study maximized the healthcare services of patients with CD through professional spatial integration. However, numerous limitations exist: (1) the timeliness of home care services; (2) the support and connection of long-term care resources; (3) the medical referral process in remote areas; (4) the integration of the information environment; and (5) expenses not covered by National Health Insurance (NHI), all impede telecare service implementation in Taiwan. However, adopting telecare services could improve healthcare availability for NCD patients, thus reducing the medical costs and improving the accessibility of healthcare resources.

### 4.3. Limitations

This study suffers from several limitations: (a) Scope of research: This study investigated in-home NCD patients that had used telecare services from a hospital in Eastern Taiwan. Due to the remoteness and cultural peculiarity of Eastern Taiwan, the results of this study may not accurately reflect other regions. (b) Sample size and selection bias: The participants were limited to patients with CD who had received telecare services from a hospital in Eastern Taiwan as such, the selection bias existed due to purposive sampling and critical case sampling due to the nature of the disease under study. (c) Geographic factors: Over half of the participants lived in mountainous areas which presented mobility and geographical constraints. Consequently, some of the participants were unable to participate in face to face interviews, and telephone communication was substituted, thus potentially affecting the survey results. (d) No pre-post surveys were done to better assess IUT. Future studies focusing on recruiting participants from several geographical areas and with multiple forms of CDs would add useful information to the telecare development data base. The mechanism of empowerment and motivation require deeper examination and an augmented questionnaire with respect to psychological focus. The NHI Research Database should be examined for historical data analyses and comparison of previous to present costs in medical care comparisons. 

## 5. Conclusions

This study examined the IUT of in-home patients with CD in Eastern Taiwan. The results indicated a high IUT and positive perception of the Telecare services from the patients. The IUT of the patients was positively correlated with self-care behaviors and physiological indicators. Telehealth is a new service designed to improve the health self-management and preventive care of patients with CD in the comfort of their home. Telehealth is not designed to replace professional medical care, but to act as an adjunct to disseminating timely health care to patients in need in a cost effective and efficient manner. At the same time, the implementation of telehealth, while reducing overall costs to NHI, will in the long term reduce the cost of health care to NHI. Telehealth appears to empower in-home CD patients to alter their behavior, as they are made aware of their health status, which enhances their intrinsic motivation to self-care.

## Figures and Tables

**Table 1:** Demographics (N = 82).

Variable	Frequency/Mean (SD)	Percentage (%)	Variable	Frequency	Percentage (%)
Sex			Area of Residence		
Male	40	48.8	Hualien City	46	56.1
Female	42	51.2	Outside Hualien City	36	43.9
Average Age (Years)–60.55 (±12.2)			Distribution of the Residences		
≤40	5	6.1	City plain region	47	57.3
41–64	52	63.4	Aboriginal mountain region	35	42.7
≥65	25	30.5	History of Diseases		
Marital Status			Hypertension	9	11.0
			Diabetes	29	35.3
Unmarried	16	19.5	Both	44	53.7
Married	66	80.5	Medication Status		
Education Level			Single Medication (antihypertensive drugs or hypo-glycemic agents)	40	48.8
High School or Lower	70	85.4	Both	42	51.2
Junior College/or Higher	12	14.6			
Employment Status					
Employed	37	45.1			
Homemaker/Retired	45	54.9			

**Table 2:** The IUT of the participants (N = 82).

Variable	Disagree Number and Percentage (%)		Neutral Number and Percentage (%)		Agree Number and Percentage (%)	
Personal benefits gained from using the telecare services						
Participating in the telecare services enabled me to control my hypertension, diabetes, and/or hyperlipidemia.	4	4.9	6	7.3	72	87.8
Participating in the telecare services enabled me to control my body weight.	11	13.4	29	35.4	42	51.2
I believe that the health records distributed through the telecare platform enhanced the doctors’ management of my health.	24	29.3	8	9.8	50	61.0
The telecare services shortened the time, I spend on acquiring medical and healthcare services.	21	25.6	14	17.1	47	57.3
The telecare services reduced my medical and healthcare expenses.	26	31.7	15	18.3	41	50.0
Activeness in uploading or in using the platform information						
I actively measured my physiological indicators by using the telecare platform.	23	28	5	6.1	54	65.9
I actively uploaded my dietary records to the telecare platform.	60	73.2	8	9.8	14	17.1
I actively uploaded my exercising records to the telecare platform.	62	75.6	9	11.0	11	13.4
I actively used the health education information provided by the telecare platform.	59	72.0	5	6.1	18	22.0
I actively discussed my health problems with my telecare case manager.	38	46.3	20	24.4	24	29.3
Individual perception of the telecare services						
The telecare services motivated me to be responsible for managing my hypertension, diabetes, and/or hyperlipidemia.	4	4.9	19	23.2	59	72
The telecare services clarified my health status.	20	24.4	5	6.1	57	69.5
I believe that the telecare services will be prevalently used in the future.	5	6.1	7	8.5	70	85.4
The telecare services should be paid for by the individuals requiring it rather than by the NHI.	43	52.4	21	25.6	18	22.0
I am willing to present my health data to the healthcare team approved by me through the telecare platform.	5	6.1	6	7.3	71	86.6

**Table 3:** Correlation between the self-care behaviors of the participants and their IUT (N = 82) via Pearson’s correlation coefficient.

Variable	Total Score for Self-Care Behaviors	Medication Compliance Behaviors	Self-Care Behaviors	Health Promotion Behaviors	Total Score for Usage Intention of Telehealth	Personal Benefits Gained from Using Telecare	Activeness in Uploading and Employing Platform Information	Individual Perception of the Telecare
Total score for self-care behaviors	1							
Medication compliance behaviors	0.511 **	1						
Self-care behaviors	0.805 **	0.297 **	1					
Health promotion behaviors	0.673 **	−0.019	0.247 *	1				
Total score for usage intention of telehealth	0.327 **	0.017	0.257 *	0.325 **	1			
Personal benefits gained from using telecare	0.247 *	−0.045	0.192	0.285 **	0.834 **	1		
Activeness in uploading and employing platform information	0.256 *	−0.043	0.203	0.289 **	0.773 **	0.485 **	1	
Individual perception of the telecare	0.279 *	0.147	0.249 *	0.160	0.789 **	0.662 **	0.489 **	1

**: Reached a statistical significance when α = 0.01 (2-tailed); *: Reached a statistical significance when α = 0.05 (2-tailed).

**Table 4:** Physiological indicators of the participants.

Indicator	*n*	Before Use of the Telecare Services	After Use of the Telecare Services	*t*	*p*
HbA1c (%)	66	8.7	8	−3.78	0.000 *
AC sugar	66	182.7	159.7	−3.14	0.003 *
Monthly frequency of BP measurement	75	3.08	6.68	4.39	0.000 *

* Reached a statistical significance when *p* < 0.05.
